# Incidence and risk factors for surgical site infection following enucleation in dogs

**DOI:** 10.3389/fvets.2022.1086956

**Published:** 2023-01-12

**Authors:** Samantha J. Dacanay, Renee M. Barber, Kathryn A. Diehl, Kathern E. Myrna

**Affiliations:** Department of Small Animal Medicine and Surgery, University of Georgia College of Veterinary Medicine, Athens, GA, United States

**Keywords:** dog, enucleation, transpalpebral, subconjunctival, surgical site infection

## Abstract

**Introduction:**

Surgical site infections (SSI) increase morbidity, increase treatment costs, and can delay onset of necessary adjunctive therapy. The goal of this retrospective study was to determine the incidence of and risk factors of SSI after enucleation in dogs.

**Methods:**

Medical records were searched at one veterinary teaching hospital and identified 280 dogs that underwent enucleation and had an adequate follow-up to assess SSI. Multiple preoperative (e.g., reason for enucleation), peri-operative (e.g., surgical approach and surgeon experience level), and post-operative (e.g., use of post-operative antibiotics or anti-inflammatory medications) variables were assessed as risk factors for development of SSI.

**Results:**

The incidence of SSI after enucleation was 5%, and no risk factors for SSI were identified. Dogs that received cephalexin as a prophylactic post-operative antibiotic were statistically more likely to develop SSI versus those that received a different post-operative antibiotic (*p* = 0.045). However, the clinical significance of this finding is unclear as administration of prophylactic post-operative antibiotics overall did not reduce the risk of SSI in the population evaluated here.

**Discussion:**

No risk factors were identified to guide clinical decision-making for prevention of SSI. Additionally, the results do not support the use of prophylactic antibiotics after enucleation in dogs.

## 1. Introduction

Enucleation is a common orbital surgery performed in dogs to remove painful, non-visual eyes or for treatment of diseases such as intraocular neoplasia that are not amenable to alternative medical or surgical therapies. Post-operative complications include hemorrhage, draining fistulas from incomplete removal of secretory tissues within the orbit, and orbital emphysema in brachycephalic breeds (1). Despite the fact that enucleation is considered a clean-contaminated procedure (2) and there are inherent challenges in maintaining effective hemostasis and minimizing dead space, surgical site infection (SSI) is considered a rare complication (1). However, SSI is still important to consider as it can increase morbidity and treatment cost and potentially lead to delays in adjunctive therapy, such as administration of chemotherapy.

SSIs following procedures without implants typically occur within 30 days after the surgical procedure and up to a year following implant placement. Criteria for diagnosis include the presence of pain, redness, swelling, heat, and purulent discharge. Additionally, if an aseptic culture is obtained, there must be growth of a microbial organism (3). The overall incidence of SSIs in dogs and cats undergoing surgical procedures in veterinary teaching hospitals has been reported to be 5.1–8.7%, with clean-contaminated procedures developing SSIs in 4.5–8.6% of cases (4, 5).

The specific incidence and risk factors associated with SSI after enucleation in dogs have not been reported. In a report of 186 dogs that had a silicone orbital implant placed after enucleation of 215 eyes, confirmed infection occurred in two eyes (1%) and cellulitis without a culture obtained occurred in an additional two eyes (6). Similarly, a study on owner perceptions of bilateral enucleation in dogs reported a 2% incidence of SSIs (7). Alternatively, in 117 cases of enucleation in horses, the rate of SSIs was 7.5%. In that study, implant placement and standing procedures were associated with increased risk of SSIs (8). The goal of this retrospective study was to determine the incidence of SSIs and risk factors for SSI following enucleation with or without implant placement in dogs. Based on anecdotal experience, the authors hypothesized infection rates would be higher than previously reported. Additionally, the authors sought to determine if there is a benefit to post-operative antibiotic administration in this specific population.

## 2. Materials and methods

### 2.1. Study population

Medical records reviewed from all dogs that underwent enucleation at the University of Georgia Veterinary Teaching Hospital from 2008 to 2022. Dogs were included if they had undergone enucleation and had at least 30 days follow-up performed by a veterinarian.

### 2.2. Data collection

The following pre-operative data were collected from the medical record: age, gender, breed, body weight, the presence of comorbidities (e.g., diabetes mellitus, hypertension), the reason for enucleation, and information regarding pre-operative administration of antibiotics. The indication for enucleation was determined and categorized as clean (phthisis bulbi, primary or secondary glaucoma, uveitis without an identified infectious etiology, anterior lens luxation, retinal disease), non-clean (perforated cornea, corneal ulcer, proptosis, retrobulbar abscess), or neoplastic. The following peri-operative information was collected from the medical record: anesthesia and surgery time, lowest systolic blood pressure, lowest body temperature, unilateral versus bilateral enucleation, surgical approach (transpalpebral vs. subconjunctival), surgeon experience level (with novice defined as intern, specialty intern, first- or second-year resident and expert defined as third-year resident or ophthalmology faculty), whether local anesthesia was administered and route of administration, whether absorbable gelatin sponge was used during the procedure, whether or not a prosthetic implant was placed, the type of suture that was used, the method of skin closure, whether concurrent ocular surgery was performed, presence of surgical complications, anesthesia and surgery time, lowest systolic blood pressure, lowest body temperature, and the type of perioperative antibiotic used. Post-operative variables recorded from the medical record included administration of systemic post-operative anti-microbial, anti-inflammatory, chemotherapeutic, and pain medications; date of follow up and diagnosis of SSI; and information regarding occurrence of an SSI, including time to infection, examination findings, culture results (when applicable), treatment, and response to treatment. Diagnosis of an SSI was made if the surgical site had evidence of pain, redness, swelling, heat, purulent discharge, persistent (>24 h) serosanguinous discharge, dehiscence, or displacement of the implant. In some cases, SSI was confirmed by aseptic culture. Cases were not considered infectious if aseptic culture results revealed no growth.

### 2.3. Statistical analysis

Association of SSI with age, gender (male vs. female), patient weight, presence of comorbidities, reason for enucleation (clean vs. not clean vs. neoplasia), length of anesthesia and surgery, presence of hypotension and hypothermia, unilateral vs. bilateral enucleation, surgical approach, level of surgeon experience, placement of a prosthetic implant, suture type, method of skin closure, presence of surgical complications, type of peri-operative antibiotic used, use of systemic post-operative antibiotics, type of post-operative antibiotic used, and use of peri- or post-operative anti-inflammatory or immunomodulatory medications was determined. Continuous data are reported as the mean ± standard deviation if normality was confirmed *via* histogram and normal quantile plot and as median (range) if not normally distributed. Categorical variables are represented as a percentage. Presence of an SSI vs. variables of interest was evaluated with univariable nominal logistic regression. When quasi-complete separation was present, Fisher's exact test was used; this occurred for the following variables: suture type; post-operative enrofloxacin, ampicillin, clindamycin, non-steroidal anti-inflammatory medication, prednisone, and chemotherapy. Statistical significance for all tests was set at *p* < 0.05. Prism 9.4.0 was used for analysis.

## 3. Results

Two hundred and eighty dogs were included in this study. The mean age of all dogs was 9.1 ± 3.7 years. There were 18 (6.4%) intact female dogs, 124 (44.3%) spayed female dogs, 23 (8.2%) intact male dogs, and 115 (41%) neutered male dogs. There were 61 breeds represented with the most common being mixed breed dog (43/280, 15.3%), shih tzu (32/280, 11.4%), Boston terrier (18/280, 6.4%), cocker spaniel (13/280, 4.6%), and Yorkshire terrier (13/280, 4.6%).

One hundred and forty-one dogs (50.4%) had the left eye enucleated, 121 dogs (43.2%) had the right eye enucleated, and 18 dogs (6.4%) had both eyes removed for a total of 298 enucleations performed. The primary indication for enucleation was associated with a clean eye in 161 dogs (57.5%), a non-clean eye in 98 dogs (35%), and neoplasia in 21 dogs (7/5%) (Table 1). Of the 98 dogs with a non-clean eye, 42 (42.9%) received pre-operative antibiotics (31/42 topical, 9/42 topical and systemic, and 2/42 systemic only). Comorbidities, including cardiac disease, kidney disease, liver diseases, m neurological disease, systemic hypertension, non-ocular neoplasia, diabetes mellitus, hypothyroidism, and hyperadrenocorticism were identified in 37/280 (13.2%) with diabetes mellitus being the most common comorbidity in 19/280 (6.8%) of dogs.

**Table 1:** Clean vs. non-clean indications for enucleation in the study cohort including percentage of overall population (*n* = 280).

**Clean (*n* = 161)**			**Non-clean (*n* = 98)**		
**Indication**	***n***	**%**	**Indication**	***n***	**%**
Secondary glaucoma	103/280	36.8	Corneal perforation	53/280	18.9
Primary glaucoma	36/280	12.9	Corneal ulceration	29/280	10.4
Uveitis	15/280	5.4	Proptosis	15/280	5.4
Anterior lens luxation	3/280	1.1	Retrobulbar abscess	1/280	0.4
Phthisis bulbi	2/280	0.7			
Retinal disease	2/280	0.7			

Dogs with neoplasia as an indication for enucleation (*n* = 21) are excluded from this table.

Surgical approach was recorded in 211 dogs with a transpalpebral approach used in 142/211 (67.3%) of dogs and subconjunctival used in 69/211 (32.7%) of dogs. For 200/280 (71.4%) of dogs, the surgeon was considered novice, while for 80/280 (28.6%) of dogs the surgeon was considered experienced. Use of local anesthesia was recorded in 141 dogs with 108/141 (76.6%) receiving a retrobulbar block and 19/141 (13.5%) receiving a splash block. Use of absorbable gelatin sponge (Gelfoam, Pfizer, New York, NY, USA) for hemostasis was recorded in 54/171 (31.6%) of dogs. Thirty-seven dogs had a concurrent ocular surgery performed, including phacoemulsification, canthoplasty, conjunctival graph placement, tarsorrhaphy, cryotherapy, and mass biopsy. Sixty-three dogs (22.5%) had a prosthetic implant placed. Information regarding the type of suture used for deep tissues and skin was recorded in 172 dogs with Polydioxanone (PDS, Ethicon, Bridgewater, New Jersey) being the most common used for deep tissues (161/172, 93.6%) and nylon (Ethilon, Ethicon, Bridgewater, New Jersey) being the most commonly used for skin closure (158/172, 91.9%). Information regarding length of anesthesia; length of surgery; and presence of hemorrhage, hypotension, and hypothermia was recorded in 275 dogs. The average duration of anesthesia for all cases was 118.4 minutes and the average duration of surgery for all cases was 63 minutes. Seventy-seven cases (28%) had intraoperative hemorrhage with 9 cases (1.2%) having lost greater than 1.3% of blood volume (9). Hypotension (systolic blood pressure less than 90 mmHg) occurred in 101 cases (36.7%) with an average lowest systolic blood pressure of 88 mmHg. Hypothermia (body temperature less than 98°F) occurred in 147 cases (53.5%) with an average lowest temperature of 97.3°F. Information on peri-operative antibiotic usage was recorded in 251 dogs. Perioperative antibiotics administered included cefazolin (233/251, 92.8%), ampicillin-sulbactam (17/251, 6.8%), and cefoxitin (1/251, 0.4%). Perioperative antibiotics were chosen based on clinician preference or aerobic culture and sensitivity results (*n* = 5) and administered 30 min prior to skin incision and every 90 min for the duration of surgery.

Two hundred and two dogs were administered systemic post-operative antibiotics based on clinician preference or aerobic culture and sensitivity results (*n* = 5), including amoxicillin-clavulanate (168/202, 83.2%), cephalexin (16/202, 7.9%), cefpodoxime (14/202, 6.9%), enrofloxacin (2/202, 1%), ampicillin (1/202, 0.5%), and clindamycin (1/202, 0.5%). Two hundred and thirty-four dogs received non-steroidal anti-inflammatory (203/234, 86.8%) or immunomodulatory (prednisone or chemotherapy) (31/234, 13.2%) medications within 2 weeks of surgery.

Time to follow-up ranged from 5 to 420 days in all dogs (median 14 days) and 5 to 420 days in dogs diagnosis with SSI (median 14 days). Fourteen dogs (14/280, 5%) developed an SSI. The median time to diagnosis of an SSI was 14 days (range 3–420). Documented clinical signs of infection included pain (8/14, 57.1%), redness (10/14, 71.4%), swelling (10/14, 71.4%), drainage from the surgical site (10/14, 71.4%) that was characterized as purulent (8/14, 57.1%) or hemorrhagic (2/14, 14.3%), dehiscence (3/14, 21.4%), or displacement of the implant (2/14, 14.3%). Five had the infection confirmed by culture with the following organisms identified: *Staphylococcus aureus*, multi-drug resistant (MDR) *Staphylococcus psuedointermedius*, MDR *Staphylococcus intermedius*, and MDR *Staphyloccosus species*.

The following were not associated with increased risk for development of an SSI: age; gender; weight; presence of comorbidities; concurrent diabetes mellitus; whether the reason for enucleation was clean, not clean, or neoplasia; administration of pre-operative antibiotics; length of anesthesia or surgery; presence of hypotension or hypothermia; unilateral or bilateral enucleation; surgical approach (transpalpebral vs. subconjunctival); experience of the surgeon; procedure for administration of local anesthesia (retrobulbar or splash block); use of absorbable gelatin sponge in surgery; concurrent ocular surgery; placement of a prosthetic implant; the type of suture used for deep tissues and skin; type of peri-operative antibiotic used; administration of post-operative antibiotics; or administration of anti-inflammatory or immunomodulatory medications.

A higher percentage of dogs that received cephalexin post-operatively developed an SSI vs. those that received other post-operative antibiotics (*p* = 0.045). However, when looking at overall post-operative antibiotic administration, dogs that received antibiotics were not less likely to develop an SSI (Table 2).

**Table 2:** Characteristics of the study cohort and results of univariable logistic regression and Fisher's exact test to assess for risk factors associated with surgical site infection after enucleation in dogs.

**Binary variables**	**No. of dogs**	**SSI**		**No SSI**		**OR (95% CI)**	***p*-value**
		***n***	**%**	***n***	**%**		
Gender (female vs. male)	280	10/14	71.4	132/266	47.1	1.8 (0.61–5.99)	0.294
Presence of comorbidities	280	3/14	21.4	46/266	17.3	1.34 (0.29–4.5)	0.671
Presence of diabetes mellitus	280	3/14	21.4	17/266	6.4	4.0 (0.86–14.31)	0.068
Reason for enucleation	280						
Clean		10/14	71.4	151/266	56.8	2.79 (0.85–12.55)	0.094
Not clean		2/14	14.3	96/266	36.1	0.31 (0.05–1.17)	0.088
Neoplasia		2/14	14.3	18/266	7.1	0.85 (0.05–4.6)	0.88
Administration of pre-operative antibiotics	280	2/14	14.3	40/266	15	0.94 (0.14–3.63)	0.938
Surgical approach (transpalpebral vs. subconjunctival)	211	7/10	70	135/201	67.2	1.0 (0.21–3.91)	>0.999
Experience of surgeon (novice versus experienced)	280	8/14	57.1	192/266	72.2	0.50 (0.17–1.58)	0.219
Local anesthetic procedure	141						
Retrobulbar		6/7	85.7	102/134	76.1	1.88 (0.31–36.2)	0.538
Splash		1/7	14.3	18/134	13.4	1.01 (0.05–6.37)	0.994
Use of absorbable gelatin sponge in surgery	171	2/8	25	52/163	31.9	0.71 (0.10–3.21)	0.683
Presence or absence of intraoperative hemorrhage	275	4/14	28.6	73/261	28	0.82 (0.22–2.54)	0.745
Intraoperative hemorrhage >1.3% blood volume	275	1/14	7.1	8/261	3.1	0.85 (0.05–4.6)	0.879
Presence of hypotension (<90 mmHg systolic blood pressure)	275	5/14	35.7	96/261	36.8	0.78 (0.24–2.35)	0.674
Presence of hypothermia (<98°F)	275	10/14	71.4	137/261	52.5	1.35 (0.45–4.49)	0.595
Concurrent ocular surgery	280	3/14	21.4	34/266	12.8	1.86 (0.41–6.33)	0.359
Placement of a prosthetic implant	280	4/14	21.4	39/266	14.7	2.33 (0.61–7.35)	0.196
Suture type deep tissues	172						
Polydioxanone (PDS)		8/8	100	153/164	95		>0.999
Polyglactin (Vicryl)		0/8	0	9/164	5.7		>0.999
Polyglecarpone (Monocryl)		0/8	0	2/164	1.3		>0.999
Suture type skin closure	172						
Nylon (Ethilon)		8/8	100	150/164	96.3		>0.999
Polypropylene (Prolene)		0/8	0	8/164	4.9		>0.999
Polydioxanone (PDS)		0/8	0	5/164	3		>0.999
Multiple		0/8	0	1/164	0.61		>0.999
Type of peri-operative antibiotic used	251						
Cefazolin		13/13	100	220/238	92.4	4.8 (0.91–88.86)	0.068
Ampicillin-sublactam		0/13	0	17/238	7.1		>0.999
Cefoxitin		0/13	0	1/238	0.4		>0.999
Administration of post-operative antibiotics	280	11/14	78.6	191/266	71.8	1.44 (0.44–6.49)	0.572
Type of post-operative antibiotic used	202						
Amoxicillin-clavulanate		7/11	63.6	161/191	84.4	0.33 (0.09–1.30)	0.103
Cephalexin		3/11	63.6	13/191	6.8	5.14 (1.03–20.32)	**0.045**
Cefpodoxime		1/11	9.1	13/191	6.8	1.37 (0.07–8.03)	0.781
Enrofloxacin		0	0	2	1		0.155
Ampicillin		0	0	1	0.5		>0.999
Clindamycin		0	0	1	0.5		>0.999
Concurrent anti-inflammatory / immunomodulatory medications	234						
Non-steroidal anti-inflammatory		13/13	100	190/221	86		0.223
Prednisone		0/13	0	28/221	12.7		0.375
Chemotherapy		0/13	0	3/221	1.3		>0.999
**Continuous variables**	**No. of dogs**	**SSI**		**No SSI**		**OR (95% CI)**	***p*** **-value**
		***n***	**Mean**	***n***	**Mean**		
Age (years)	280	14	9.2	266	9.1	1.01 (0.88–1.18)	0.888
Weight (kg)	280	14	11.2	266	14	0.98 (0.91–1.03)	0.385
Duration of anesthesia (min)	275	14	123.4	261	118.1	1.0 (0.99–1.0)	0.668
Duration of surgery (min)	275	14	62.4	261	63	0.99 (0.97–1.01)	0.943
Lowest systolic blood pressure during surgery (mmHg)	275	14	85.7	261	88.3	0.992 (0.96–1.02)	0.589
Lowest temperature during surgery (°F)	275	14	97.1	261	97.3	0.92 (0.71–1.23)	0.568

Continuous variables were all normally distributed and are reported as mean ± standard deviation. For dichotomous variables, the variable of interest is either yes vs. no or is reported in parentheses after the variable name.

No, number; SSI, surgical site infection; OR, odds ratio; CI, confidence interval.

## 4. Discussion

In the retrospective study reported here, the overall incidence of SSI following canine enucleation was 5%. This is similar to previously reported infection rates of 4.5–8.6% for clean-contaminated surgeries performed in veterinary teaching hospitals (4, 5) and similar to a reported SSI incidence of 7.5% in horses undergoing transpalpebral enucleation (8). However it is higher than two canine enucleation studies that reported an SSI incidence of <2 (6, 7). The SSI incidence of 5% reported here may be an overestimation. Four hundred eleven cases of enucleation were identified in the medical records search but only 280 had adequate follow-up information to allow inclusion. It is possible that the dogs without follow-up did not return to a veterinarian because they did not have any complications. Equally, only 5/14 (35.7%) of cases had confirmation of infection by culture so it is possible that the reported 5% is an overestimation.

Numerous previously reported SSI-risk factors, including pre-operative variables such as reason for enucleation; peri-operative variables such as surgical approach, surgeon experience, and surgical approach; as well as post-operative variables such as use of post-operative antibiotics or anti-inflammatory medications were assessed in the patient population presented here. Overall, no risk factors for development of SSI after enucleation were identified. The only finding that reached statistical significance was that dogs administered cephalexin post-operatively were more likely to develop an infection than those that received other post-operative antibiotics. However, the clinical significance of this finding is unclear. First, it is important to note that administration of post-operative antibiotics regardless of type did not reduce the risk of SSI. Additionally, a large number of variables were assessed in this study making it possible that this significant finding occurred due to chance. The authors do not believe this finding should change clinical decisions made for canine patients undergoing enucleation.

In equine patients undergoing enucleation, standing surgery and implant placement were associated with increased risk of SSI. All patients in our population were placed under general anesthesia for enucleation, and, although a minority had implants placed, implant placement was not associated with an increased risk for infection. It is possible that with longer follow-up times of at least a year, additional SSIs in the dogs that had implants placed would have been identified. Ultimately, a prospective study with larger case numbers may help identify risk factors associated with enucleation in dogs.

One impetus for this retrospective study was to determine if post-operative antibiotics reduce the risk of SSI following enucleation in dogs. As mentioned, administration of post-operative antibiotics did not decrease the risk of SSI in this study. This is consistent with a metaanalysis in people of 52 randomized controlled trials with 19,273 participants that did not find conclusive evidence that prophylactic post-operative antibiotics reduce the risk of SSI (10). Additionally, although only a small number (*n* = 5) of cases reported here had positive bacterial cultures, 60% (3/5) grew multi-drug resistant (MDR) organisms. This is in line with reports of human SSI where 37–65% of isolated organisms have a MDR profile (11, 12). Based on the fact that post-operative antibiotics did not reduce the risk of SSI and can increase the risk of development of MDR pathogens (13, 14), prophylactic post-operative use of antibiotics following enucleations in dogs is not supported.

There are several limitations to this study. It is retrospective in nature so some information in the medical records was incomplete and as a result certain previously reported risk factors such as increased duration of anesthesia and surgery as well as intraoperative hypotension could not be assessed (15–19). Although the study included a large number of dogs overall, only a small number of cases had SSI, which may have influenced our ability to identify risk factors. Not all cases of enucleation for the time period of the retrospective study were able to be included because they lacked adequate follow-up information; this may have skewed the SSI incidence and ability to identify risk factors. Also, as previously mentioned, not all cases of infection were confirmed with aerobic culture, which might have influenced SSI incidence.

Based on this report, risk of SSI after enucleation in dogs is 5%. No risk factors were identified to guide clinical decision-making for prevention of SSI. However, lack of confirmation that systemic post-operative antibiotics reduce SSI risk in combination with concerns about increasing numbers of MDR pathogens involved in SSI, indicates prophylactic antibiotics should not be used be after surgery in routine cases of canine enucleation.

## Data availability statement

The original contributions presented in the study are included in the article/supplementary material, further inquiries can be directed to the corresponding author.

## Author contributions

SD was responsible for data acquisition. SD and RB were responsible for data analysis. All authors were responsible for study design, manuscript preparation, and approved the submitted version of the manuscript.
